# Exploration of microRNAs in porcine milk exosomes

**DOI:** 10.1186/1471-2164-15-100

**Published:** 2014-02-05

**Authors:** Ting Chen, Qian-Yun Xi, Rui-Song Ye, Xiao Cheng, Qi-En Qi, Song-Bo Wang, Gang Shu, Li-Na Wang, Xiao-Tong Zhu, Qing-Yan Jiang, Yong-Liang Zhang

**Affiliations:** Guandong Provincial Key Lab of Agro-Animal Genomics And Molecular Breeding, College of Animal Science, ALLTECH-SCAU Animal Nutrition Control Research Alliance, National Engineering Research Center For Breeding Swine Industry, South China Agricultural University, Guangzhou, 510642 China

**Keywords:** Porcine milk exosomes, Solexa sequencing, miRNA

## Abstract

**Background:**

Breast milk contains complex nutrients and facilitates the maturation of various biological systems in infants. Exosomes, membranous vesicles of endocytic origin found in different body fluids such as milk, can mediate intercellular communication. We hypothesized that microRNAs (miRNAs), a class of non-coding small RNAs of 18–25 nt which are known to be packaged in exosomes of human, bovine and porcine milk, may play important roles in the development of piglets.

**Results:**

In this study, exosomes of approximately 100 nm in diameter were isolated from porcine milk through serial centrifugation and ultracentrifugation procedures. Total RNA was extracted from exosomes, and 5S ribosomal RNA was found to be the major RNA component. Solexa sequencing showed a total of 491 miRNAs, including 176 known miRNAs and 315 novel mature miRNAs (representing 366 pre-miRNAs), which were distributed among 30 clusters and 35 families, and two predicted novel miRNAs were verified targeting 3’UTR of IGF-1R by luciferase assay. Interestingly, we observed that three miRNAs (ssc-let-7e, ssc-miR-27a, and ssc-miR-30a) could be generated from miRNA-offset RNAs (moRNAs). The top 10 miRNAs accounted for 74.5% (67,154 counts) of total counts, which were predicted to target 2,333 genes by RNAhybrid software. Gene Ontology and Kyoto Encyclopedia of Genes and Genomes (KEGG) pathway analyses using DAVID bioinformatics resources indicated that the identified miRNAs targeted genes enriched in transcription, immunity and metabolism processes, and 14 of the top 20 miRNAs possibly participate in regulation of the IgA immune network.

**Conclusions:**

Our findings suggest that porcine milk exosomes contain a large number of miRNAs, which potentially play an important role in information transfer from sow milk to piglets. The predicted miRNAs of porcine milk exosomes in this study provide a basis for future biochemical and biophysical function studies.

**Electronic supplementary material:**

The online version of this article (doi:10.1186/1471-2164-15-100) contains supplementary material, which is available to authorized users.

## Background

Milk, as the sole source of nutrition for infants, contains a potent mixture of diverse components such as milk fat globules (MFG) [1], immune competent cells and soluble proteins, for instance IgA, cytokines and antimicrobial peptides [2], which can provide protection against infections in newborns [3]. In addition, breast milk may have a role in tolerance induction [1] and may protect infants from developing allergies [4].

Exosomes are small (30–100 nm) membrane vesicles of endocytic origin that are released into the extracellular environment upon fusion of multivesicular bodies (MVB) with the plasma membrane [5]. Many cells have the capacity to release exosomes, including reticulocytes [6], dendritic cells [7], B cells [8], T cells [9], mast cells [10], epithelial cells [11] and tumor cells [12]. In addition, exosomes have been found in physiological fluids, such as saliva [13, 14], plasma [15], urine [16], amniotic fluid [17], malignant ascites [18], bronchoalveolar lavage fluid [19] and synovial fluids [20]. Several studies have suggested that exosomes, which contain proteins, mRNA and microRNA (miRNA), stimulate and transfer surface receptors to target cells [21–23], as well as serve as novel vehicles for genetic exchange between cells [24]. As with other biological fluids, microvesicle-like particles are also present in mouse milk [25] and human milk [26]. Recent published studies have isolated mRNAs and miRNAs from bovine milk-derived microvesicles [27]. One study via deep sequencing technology identified 602 unique miRNAs originating from 452 miRNA precursors (pre-miRNAs) in human breast milk exosomes and found that, out of 87 well-characterized immune-related pre-miRNAs, 59 (67.82%) were enriched in breast milk exosomes [28]. Recently, porcine milk was reported to contain 180 pre-miRNAs, including 140 known and 40 novel porcine pre-miRNAs, altogether encoding 237 mature miRNAs [29].

MiRNAs are widespread among eukaryotes and represent key components of a conserved system of RNA-based gene regulation [30–33]. Many studies have demonstrated that miRNAs are key post-transcriptional regulators of gene expression and play important roles in a wide range of physiological and pathological processes [34], including development, differentiation, proliferation and immune responses. It is believed that about 60% of mammalian genes are regulated by miRNAs [35–39].

Aside from being important farm livestock, pigs are also model animals for medical research. In the present study, we investigated miRNAs in milk exosomes of Landrace pigs in order to provide new information for investigations into the physiological functions of porcine milk.

## Methods

### Milk samples

Porcine milk samples were collected between day 1 to 5 after parturition from healthy lactating Landrace female pigs bred in the breeding farm of the Livestock Research Institute (Guangzhou, China). Milk samples were frozen immediately after milking and were kept at-80°C until use.

### Preparation of exosomes from milk

Porcine milk samples were centrifuged first at 2,000 × *g* for 30 min at 4°C to remove MFGs as well as mammary gland-derived cells. Defatted samples were then subjected to centrifugations at 4°C for 30 min at 12,000 × *g* to remove residual MFGs, casein and other debris. Subsequently, from the final supernatant (so-called whey or milk serum), the membrane fraction was prepared by ultracentrifugation at 110,000 × *g* for 2 h in an SW41T rotor (Beckman Coulter Instruments, Fullerton, CA) [40].

### Transmission electron microscopy (TEM)

The final fraction obtained as described above was diluted with 0.01 M PBS and ultracentrifuged again to recover microvesicles as pellets. Following fixation in 2% glutaraldehyde, microvesicles were negatively stained with uranyl acetate and observed by TEM (JEOL JEM2000EX, Tokyo, Japan).

### RNA isolation and Solexa sequencing

Total RNA was isolated from samples collected after ultracentrifugation using Trizol reagent (Invitrogen, Carlsbad, CA) according to the manufacturer’s protocol. The quality of RNA was examined by 2% agarose gel electrophoresis and with a Biophotometer 6131 (Eppendorf, Germany), as well as further confirmed by using a Bioanalyzer (Agilent Technologies, Santa Clara, CA). Small RNAs (18–30 nt) were obtained from the total RNA, 5’ and 3’ adaptors were ligated to the small RNAs, then the adaptor-ligated RNAs were subsequently transcribed into cDNA by RT-PCR, and the samples were amplified by PCR using primers complementary to the two adaptors. The PCR products were purified and subjected to Solexa sequencing (Illumina, CA) at the Beijing Genomics Institute (BGI, Shenzhen, China).

### Sequence data analysis

The raw reads obtained from Solexa sequencing were processed to obtain clean reads by summarizing data production, evaluating sequencing quality, calculating the length distribution of small RNA reads, removing low quality reads and adaptor sequences as described in previous paper [41]. All the clean reads were aligned against non-coding RNAs from the GenBank and Rfam (11.0) (ftp.sanger.ac.uk/pub/databases/Rfam) database to annotate and classify rRNA, tRNA, snRNA and other ncRNA sequences using tag2 annotation software (developed by BGI). Then the selected sequences were mapped to the pig genome (sscrofa9, http://www.ensembl.org/Sus_scrofa/) using SOAPv1.11 software [42] to analyze their expression and distribution. Subsequently, the miRNA candidates were further analyzed by miRDeep 2 against all known miRNAs and porcine miRNA precursors (miRBase 20.0). All remaining candidates who did not map to any miRNAs in miRBase 20.0 were considered as potential novel miRNAs. To further identify these potential novel miRNA candidates, software MIREAPv0.2 (http://sourceforge.net/projects/mireap) [43] developed by BGI was used to predict novel miRNA by exploring the secondary structure, the Dicer cleavage site and the minimum free energy of the annotated small RNAs which could be mapped to genome. In briefly, the sequence length should be between 18–26 nt, maximal free energy allowed for a miRNA precursor was -18 kcal/mol, maximal space between miRNA and miRNA* was 35 nt, and flank sequence length of miRNA precursor should be 10 nt. Finally, all remaining novel miRNA candidates were further subjected to MiPred (http://www.bioinf.seu.edu.cn/miRNA/) to filter out pseudo-pre-miRNAs. The minimum free energy must be > -20 kcal/mol or P-value was >0.05 [44], and their secondary structures were also checked using the Mfold3.2 software [45]. All data for analysis in this study have been deposited in https://mynotebook.labarchives.com/share/allinchen/MTkuNXwxMzMxMS8xNS0yL1RyZWVOb2RlLzE1NzEyODU2fDQ5LjU= with a DOI:10.6070/H4DN432G.

### PCR and qRT-PCR identification of known and novel miRNAs

Total RNA (identical sample to that of the Solexa sequencing sample) was first digested with DNase I (Invitrogen), and 2 μg of total RNA was reverse transcribed to poly (A) tail-added cDNA using the One Step PrimeScript miRNA cDNA Synthesis Kit (TaKaRa, Dalian) according to the manufacturer’s instructions. Briefly, a 60-nt adaptor containing a poly (A) structure was added to the 3’ sequence of miRNAs, which were then reverse transcribed to an 80-bp cDNA sequence [46]. The cDNA was diluted 5-fold with ddH_2_O, and PCR was performed on a Bio-Rad system (BIO-RAD,USA )in a final 20 μL volume reaction containing 2 μl PCR cDNA, 10 μL of 2× PCR Mix (Toyobo, Osaka, Japan) and 1 mM of each primer. The real-time PCR thermal profile was as follows: 5 min at 95°C, 40 cycles of 30 s at 94°C, 30 s at the corresponding annealing temperature (Tm) and 72°C for 30 s, followed by 72°C at 10 min. PCR products were examined on an agarose gel to confirm that a single PCR product was generated, and 5S ribosomal RNA was used as an internal control for the PCR. The miRNA forward primer was designed with Primer 5.0 ( Table 1), and the reverse primer for miRNAs was the Uni-miR qPCR Primer offered by the kit One Step PrimeScript miRNA cDNA Synthesis Kit (TaKaRa, Dalian).

**Table 1:** **PCR primers for miRNAs**

miRNAs name	Primer sequence	Renaturation temperature
ssc-let-7e	TGAGGTAGGAGGTTGTATAGTT	59.5°C
ssc-miR-21	GCTAGCTTATCAGACTGATGTTG	59.5°C
ssc-miR-206	TGGAATGTAAGGAAGTGTGTG	59.5°C
ssc- let-7i	GCCGCTGAGGTAGTAGTTTGTGCT	59.5°C
ssc-miR-140	GACAGTGGTTTTACCCTATGGTA	59.5°C
ssc-miR-92b-5p	TTATAGGGACGGGACGCGGTG	59.5°C
ssc-miR-22b-3p	AAGCTGCCAGTTGAAGAACTG	59.5°C
ssc-miR-28-5p	GAAGGAGCTCACACTCTATTGA	59.5°C
ssc-miR-205	TCCTTCATTCCACCGGAGTCT	59.5°C
ssc-miR-451	AAACCGTTACCATTACTGAGTT	59.5°C
ssc-miR-125b	TCCCTGAGACCCTAACTTGTG	59.5°C
ssc-miR-9	GCGGTCTTTGGTTATCTAGCTGT	59.5°C
ssc-let-7c	TGAGGTAGTAGGTTGTATGGT	59.5°C
P-m0281-5p(PS-16)	TCTCCCAACCCTTGTACCA	58°C
P-m0124-3p(PC-280)	TGTTCCGAGATTGGGCTG	58°C
P-m0227-5p(PC-291)	TTCCTGAGTCGGACTGGG	58°C
P-m0355-5p(PC-82)	CCCAGGATCAGAGGATGG	58°C
P-m0338-3p(PC-241)	TCTGTGAACTAGAAACCTCTGG	58°C
P-m0105-3p(PC-72)	CATTTGATTCAGTTGGACACT	58°C
P-m0113-3p(PC-130)	CTATGGATCTAGGAGGACGC	58°C
P-m0129-5p(PC-129)	CTATGGATCTAAGAGGACACCC	58°C
P-m0058-5p(PC-276)	TGTGTGTGATCGTTAATGTGC	58°C
P-m0279-5p(PC-192)	GTCCTTGGTGAGTCGGATG	58°C
P-m0103-3p(PC-70)	CATTGCTTTGATCGTCTGG	58°C
P-m0265-3p(PC-139)	CTGGAAGGATTTGGGTAGG	58°C
P-m0210-5p(PC-277)	TGTGTGTTCTGTCGGATGAG	58°C
P-m0186-5p(PC-73)	CATTTTAAGGATCGTGTGGG	58°C
P-m0070-3p(PC-63)	CAGTAGGGATGAGAGGACACT	58°C

### miRNAs target prediction and plasmid construction

**Figure 1:**
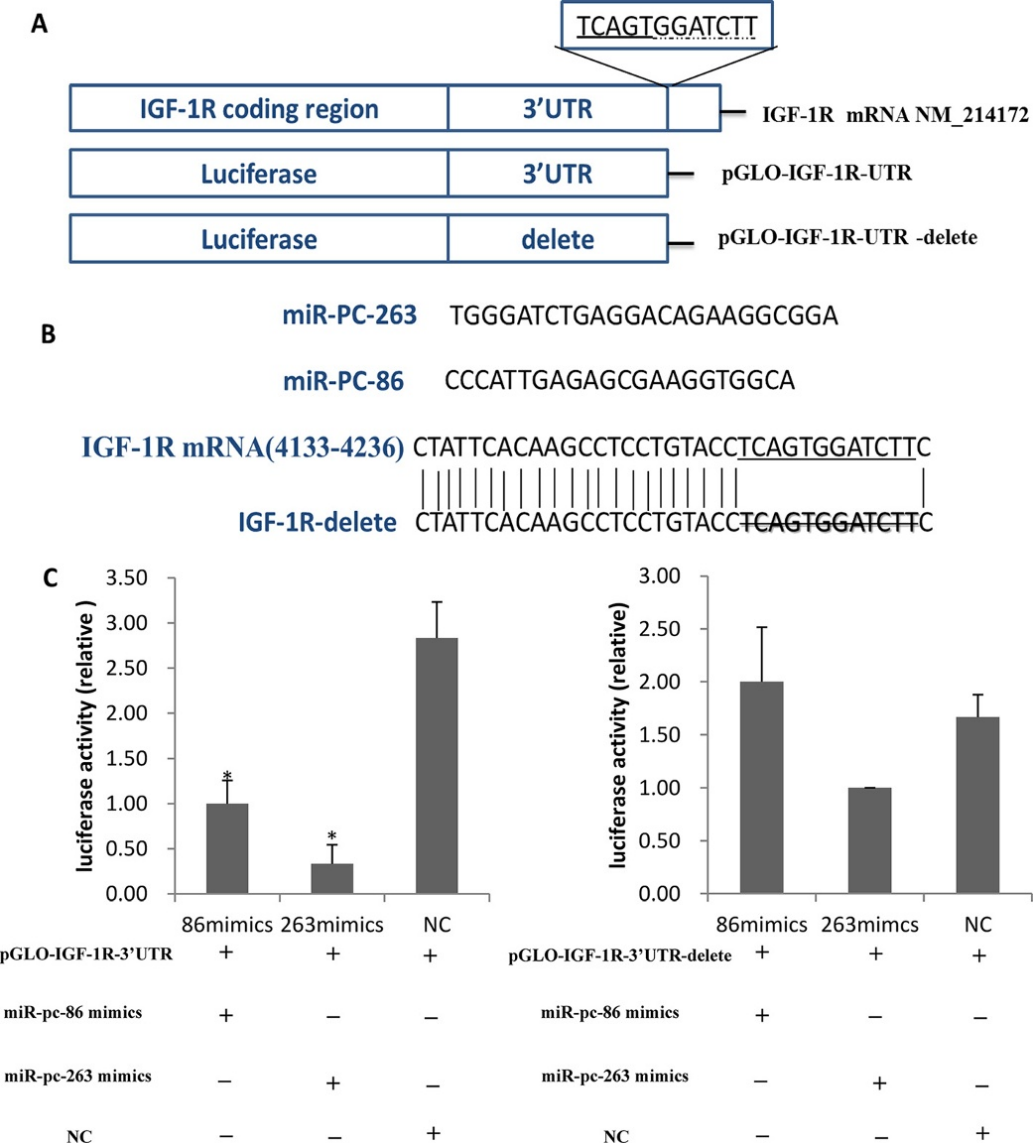
**MiR-PC-86 and MiR-PC-263 directly regulate IGF-1R expression via 3’ UTR sites. (A)** Schematic of *IGF-1R* mRNA and the luciferase reporter plasmids containing the miR-PC-86 and miR-PC-263 binding sites of *IGF-1R* mRNA. The 3’ UTR sites were inserted downstream of the luciferase reporter, as indicated. TCAGTGG was the predicted target site of miR-PC-86, GGATCTT was the predicted target site of miR-PC-263*.* **(B)** miR-PC-86 and miR-PC-263 sequences and predicted binding site between miR-PC-86 and miR-PC-263 and *IGF-1R* mRNA. *IGF-1R* mRNA has one putative binding site for miR-PC-86/ miR-PC-263 on the 3’ UTR. Twelve nucleotides TCAGTGGATCTT of *IGF-1R* 3’ UTR (underlined) were delete in order to disrupt the binding with miR-PC-86 and miR-PC-263 seed regions. **(C)** IPEC-J2 cells were transfected with each of the constructed plasmids, together with miR-PC-86/ miR-PC-263and Renilla luciferase reporter plasmid (*P < 0.05, n = 6).

Two predicted novel miRNAs, named miR-PC-86 and miR-PC-263, were selected to predict their target genes in pig genome using the RNAhybrid software (http://bibiserv.techfak.uni-bielefeld.de/rnahybrid/) with its own algorithm. The 3’-UTR sequences of porcine transcripts in whole genome were obtained from ensemble gene 66 (sscorfa 9, http://www.ensembl.org/Sus_scrofa/). The 3’-UTR of IGF-1R contains the highly conserved binding sites for the two miRNAs, and the sequence (104 bp) is as follows:*TCCTGGATCCCGATCCCGTGCAAACAGTACCGTGCGCACGCGGGCGGGCGGGGGGAGAGTTTTAACAATCTATTCACAAGCCTCCTGTACCTCAGTGGATCTTC*. Further, the 3’-UTR sequence was inserted into pmirGLO Vector (Promega) with *XhoI* and *XbaI* double digestion to construct recombinant Dual-Luciferase reporter vector, named as pGLO-IGF-1R-3’UTR (Figure 1A). Meanwhile, a plasmid containing mutant IGF-1R 3′-UTR, named as pGLO-IGF-1R-3’UTR-delete (Figure 1A), was generated by deleting the core sequence of the two miRNA binding sites through DNA synthesis (Sangon Biotech (Shanghai) Co., Ltd.), the sequence is as follows: *TCCTGGATCCCGATCCCGTGCAAACAGTACCGTGCGCACGCGGGCGGGCGGGGGGAGAGTTTTAACAATCTATTCACAAGCCTCCTGTACCC*.

### Leuciferase reporter assay

IPEC-J2 cells were maintained in DMEM/F12 (1:1) (GIBCO) and supplemented with 10% fetal bovine serum (FBS, GIBCO), 5 ng/ml EGF (peprotech, USA) and 5 ug/ml insulin (Sigma, USA). Lipofectamine 2000 (Invitrogen) was used for transfection. Cells (10,000) were plated in a 96-well plate. After 24 h cultivation, cells were transfected with a mixture including 500 ng pGLO-IGF-1R-3’UTR or pGLO-IGF-1R-3’UTR-delete construct and 30pM of miR-PC-86 or miR-PC-263 mimics (GenePharma, Shanghai, China). For control, 500 ng of pmirGLO-scramble including a scrambled sequence of the miRNA target sequence was used. Cells were collected 48 h after transfection, and luciferase activity was measured using a Dual-GLO luciferase reporter assay system (Promega). Statistical differences between treatment and control groups were determined using Student’s t-test, at *P* < 0.05.

### Bioinformatics analysis

#### Chromosomal localization and cluster analysis of miRNAs

Pre-miRNAs of all miRNAs (known miRNAs and novel miRNAs) were mapped to the porcine genome (sscrofa9, http://www.ensembl.org/Sus_scrofa/) according to their positions on the chromosomes. Pre-miRNA positions less than 10 kb apart were considered to belong to the same miRNA cluster.

### Target prediction and Gene Ontology (GO) and Kyoto Encyclopedia of Genes and Genomes (KEGG) pathway analyses

Porcine miRNA targets were obtained at the genome level. In brief, miRNA targets were predicted using the RNAhybrid software algorithm (http://bibiserv.techfak.uni-bielefeld.de/rnahybrid/) in 3’-UTR sequences of transcripts from the whole pig genome obtained from Ensembl Gene 66 database (sscrofa9, http://www.ensembl.org/Sus_scrofa/). Strict criteria (perfect match of 2–8 nt in the seed region; no more than -25 kcal/mol low free energy of miRNAs-mRNA binding) were applied to the target prediction procedure. GO and KEGG pathway analyses were performed using DAVID bioinformatics resources (http://david.abcc.ncifcrf.gov/).

## Results

### Identification of exosomes

**Figure 2:**
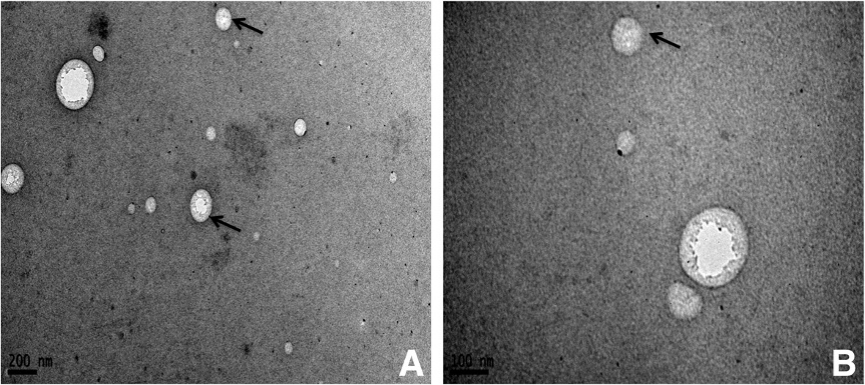
**Exosomes detected by TEM.** The exosomes appeared as round or oval microvesicles **(A, B)**, with a diameter of 50–150 nm and heavier density at the center than on the margin.

Exosomes were obtained from porcine milk by ultracentrifugation. After negative staining, approximately round-shaped porcine milk exosomes with diameters of ~50-100 nm were observed by TEM, showing a greater density at the center than at the boundary (Figure 2A, B).

### Porcine milk exosomes contain RNA

**Figure 3:**
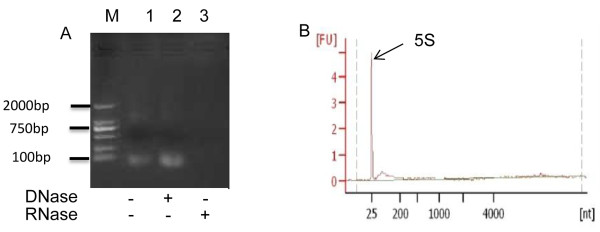
**Milk-derived exosomes containing RNA. (A)** Total RNA was extracted from porcine exosomes. M, 1, 2 and 3 represent the marker (DL 2000), RNA without any treatment, RNA treated with DNase I and RNA treated with RNase, respectively. **(B)** RNA sample analyzed by the Agilent Bioanalyzer 2100.

To further ascertain whether the porcine milk exosomes collected by ultracentrifugation contained RNA, we extracted the samples using Trizol reagent and then examined the recovered product by electrophoresis on a 2% agarose gel. To exclude the possibility of DNA contamination, total RNA was incubated at 37°C for 30 min with DNase I or RNase. The extracted RNA could not be digested by DNase I, while it could be degraded by RNase (Figure 3A). These results demonstrated that the porcine milk exosomes contained RNA. More interestingly, total RNA of porcine milk exosomes were enriched with 5S rRNA (Figure 3B), consistent with previous studies [27, 28, 47].

### Solexa sequencing and analysis

#### Solexa sequencing

**Figure 4:**
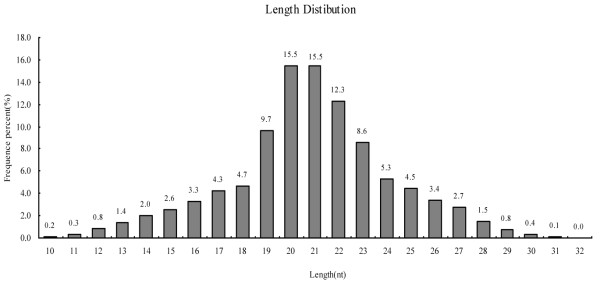
**Length distribution of miRNAs reads from Solexa sequencing.** A total of 4,964,542 clean reads were obtained, ranging from 10 to 32 nt, most of which were 18–25 nt in length (accounting for 74.89%).

The sRNAs were enriched from porcine exosomes to construct a library for Solexa sequencing. We obtained 9,033,167 raw reads and 6,013,724 high qualities reads after removal of low quality and contaminant reads. Among the high quality reads, there were 4,964,542 clean reads (82.55%), representing 1,691,655 unique sRNAs. The majority of the sRNAs in porcine milk exosomes were 18–25 nt in length (74.89%, Figure 4), with 2,458,894 reads (49.53%) representing 872,096 unique sRNAs (51.55%), including miRNAs and other sRNAs, such as rRNA, tRNA, snRNA, snoRNA, scRNA, small recognition particle RNA (srpRNA), repetitive sequence elements and unannotated sequences, which could be mapped to the pig genome. BLAST searching with the miRbase 20.0, identified a total of 230,216 reads, representing 1,555 unique known miRNAs. Due to RNA bias editing, 5’ modifications and 3’ modifications, many pre-miRNAs produce multiple mature miRNA isoforms, namely isomiRNAs, as described in many studies [48–50]. In the subsequent analysis, all isomiRNAs generated from the same precursor were considered one type of miRNA. Consequently, these 1,555 unique miRNAs corresponded to 176 known mature miRNAs (205 pre-miRNAs, all the detail were listed in Additional file 1: Table S1). In addition, we identified 315 novel mature miRNAs (generated from 366 pre-miRNAs, detail in Additional file 2: Table S2). Among the 315 novel miRNAs, 18 have not been deposited as porcine miRNAs in miRbase 20.0, but had very high similarity with miRNA sequences of other species. These 18 miRNAs are labeled “PS” (Table 2), while 298 miRNAs that have not been deposited in miRbase 20.0 for any organism are labeled as “PC” and presented in Additional file 2: Table S2.

**Figure 5:**
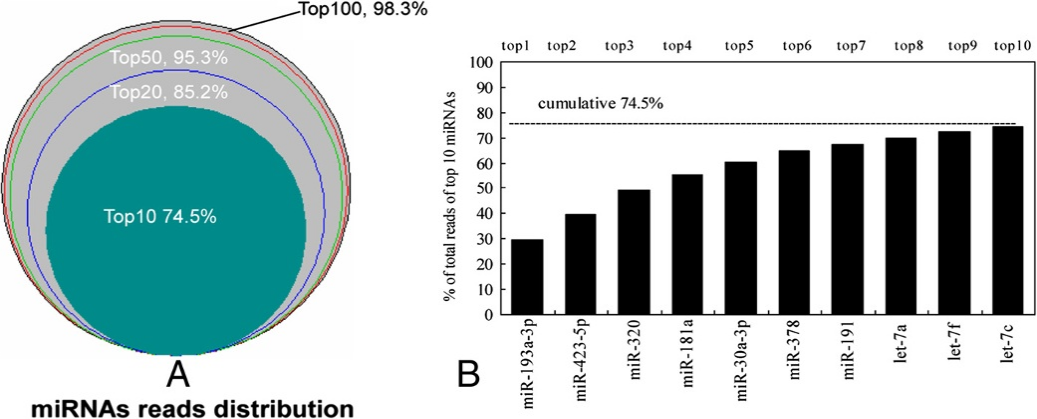
**Distribution of miRNA reads and top 10 miRNAs. (A)** The distribution of miRNA reads showed that the top 10, top 20, top 50 and top 100 miRNAs accounted for 74.5%, 85.2% and 95.3% and 98.3% of total reads. **(B)** Cumulative proportions of top 10 miRNAs. MiR-193-3p ranked first, accounting for 29.6% of total reads.

There were 73 miRNAs with more than 100 counts and 264 miRNAs with less than 10 counts. The top 10, top 20, top 50 and top 100 miRNAs accounted for 74.5%, 85.2%, 95.3% and 98.3% of the reads, respectively (Figure 5A). The top 10 miRNAs were ssc-miR-193a-3p, ssc-miR-423-5p, ssc-miR-320, ssc-miR-181a, ssc-miR-30a-3p, ssc-miR-378, ssc-miR-191, ssc-let-7a, ssc-let-7f and ssc-let-7c. With 67,154 counts (29.6%, average count: 460.6) (Figure 5B), ssc-miR-193a-3p ranked first among all miRNAs reads.

**Table 2:** **Porcine novel miRNAs conserved in other species (miRBase release 20.0)**

Unique ID	miRNAs name	Count	Sequence	Size	Conservation	Match
PS-1	miR-290-5p	14	ACTCAAACTGTGGGGGCACTTT	22	mmu(#)	1nt sub(#)
PS-2	miR-378c	33	ACTGGACTTGGAGTCAGAAGT	21	hsa	4nt delete
PS-3	miR-20b-3p	4	ACTGTAGTGTGGGCACTTCCAGT	23	hsa	1nt add, 1nt sub
PS-4	miR-219-3p	11	AGAATTGTGGCTGGACATCT	20	bta	1nt delete
PS-6	miR-138-5p	42	AGCTGGTGTTGTGAATCAGGCCG	23	mmu	perfect
PS-7	miR-31-5p	5	AGGCAAGATGCTGGCATAGCT	21	has	perfect
PS-9	let-7f-1-3p	2	CTATACAATCTATTGCCTTCC	21	rno	perfect
PS-11	miR-874-3p	25	CTGCCCTGGCCCGAGGGACCGA	22	mmu	perfect
PS-12	miR-551a	225	GCGACCCACTCTTGGTTTCC	20	hsa	1nt delete
PS-13	miR-138-3p	1	GCTACTTCACAACACCAGGGT	21	hsa	1nt sub, 1delete
PS-14	miR-182-3p	1	GGTGGTTCTAGACTTGCCAACT	22	mmu	1nt insert
PS-15	miR-5003-3p	58	TATTTAATAGGTTGTTGGGA	20	hsa	2nt sub, 2nt delete
PS-16	miR-150-5p	164	TCTCCCAACCCTTGTACCAGT	21	mmu	1nt delete
PS-17	miR-2411-3p	7	TGAACTGTCATACTCCCACATC	22	bta	3nt delete, 1nt sub
PS-18	let-7f-5p	20	TGAGGTAGTAGATTGTATAGTTG	23	hsa	1nt insert
PS-19	miR-31-3p	3	TGCTATGCCAACATATTGCCA	21	has	1nt delete
PS-20	miR-182	46	TTTGGCAATGGTAGAACTCACA	22	dre	perfect
PS-21	miR-96-5p	65	TTTGGCACTAGCACATTTTTGCT	23	hsa	perfect

#: mmu, Mus musculus; hsa, Homo sapiens; bta, Bos taurus; dre, Danio rerio; rno, Rattus norvegicus;

“sub”, “delete”, “add” represents nucleotide substitution, deletion, addition (at 5-end of miRNAs), respectively perfect stands for perfect match with reference miRNAs.

### Identification of miRNAs by PCR and sequencing

**Figure 6:**
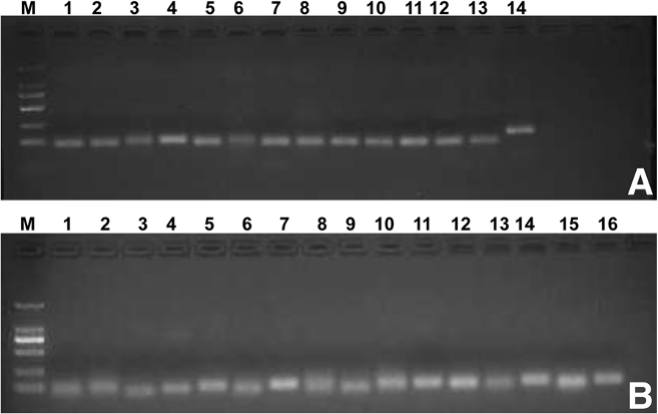
**MiRNAs detected randomly in porcine milk. (A)** Known miRNAs from miRBase (18.0), from M to 14, respectively: marker (DL 2000), ssc-let-7e, ssc-miR-21, ssc-miR-206, ssc-let-7i, ssc-miR-140, ssc-miR-92b-5p, ssc-miR-22-3p, ssc-miR-28-5p, ssc-miR-205, ssc-miR-451, ssc-miR-125b, ssc-miR-9, ssc-let-7c and 5 s (control). **(B)** Top 15 predicted novel miRNAs, from M to 16, respectively: marker (DL 2000), P-m0227-5p, P-m0338-3p, P-m0105-3p, P-m0058-5p, P-m0281-5p, P-m0265-3p, P-m0279-5p, P-m0103-3p, P-m0113-3p, P-m0129-5p, P-m0355-5p, P-m0210-5p, P-m0070-3p, P-m0124-3p, P-m0186-5p and 5 s (control).

**Figure 7:**
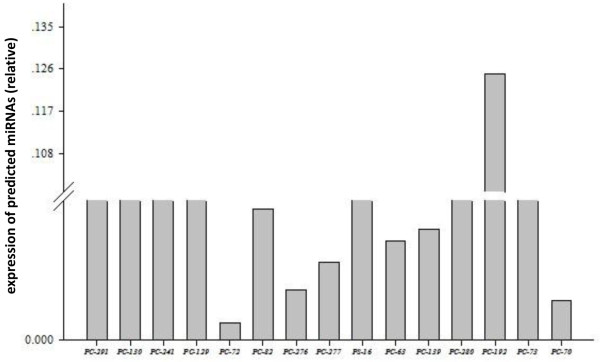
**Expression of 15 predicted novel miRNAs in the sample pool detected by qRT-PCR.** Trends in relative expression by qRT-PCR and counts from Solexa sequencing of miRNAs, except for PC-192, were consistent.

To verify the deep sequencing results, we selected randomly 13 known miRNAs and 15 predicted novel miRNAs for PCR amplification (Figure 6A, B). Subsequently, the 15 newly predicted miRNAs were cloned and sequenced. The results showed that 8 sequences were fully matched, while 7 sequences had one or two mismatched nucleotides (Table 3). However their seed sequences remained unchanged. Simultaneously, the abundance of some novel miRNAs predicted by Solexa sequencing was confirmed by quantitative real-time PCR. The abundance of most miRNAs observed by qPCR of the sample pool and by sequencing were generally consistent (Figure 7).

**Table 3:** **miRNAs matched to sequecing**

Predict new miRNA	Matched sequence
P-m0281-5p	
P-m0124-3p	
P-m0227-5p	
P-m0355-5p	
P-m0338-3p	
P-m0105-3p	
P-m0113-3p	
P-m0129-5p	
P-m0058-5p	
P-m0279-5p	
P-m0103-3p	
P-m0265-3p	
P-m0210-5p	
P-m0186-5p	
P-m0070-3p	

### Target verification of miR-PC-86 and miR-PC-263 against 3’UTR of IGF-1R using luciferase report assay

To investigate whether the predicted miR-PC-86 and miR-PC-263 (Figure 1) were functional novel miRNAs, target genes were predicted, and miR-PC-86/ miR-PC-263 were found to directly target IGF-1R 3’UTR sequence. The full-length 3’UTR of IGF-1R mRNA was inserted downstream of the luciferase gene in the pmirGLO Dual-Luciferase miRNA Target Expression Vector reporter plasmid, and the seed sequence was also delete to disrupt miR-PC-86/ miR-PC-263 binding (Figure 1B). The wild-type (pGLO-IGF-1R-3’UTR) or delete (pGLO-IGF-1R-3’UTR-delete) plasmid was co-transfected with the miR-PC-86 and miR-PC-263 mimics into IPEC-J2 cells. Forty-eight hours after transfection, the luciferase activity of the miR-PC-86 and miR-PC-263 group were significantly lower than that of the NC group (P < 0.05) respectively, and the reduction was rescued in the delete group (Figure 1C). Thus, IGF-1R was initially confirmed as the target of miR-PC-86 and miR-PC-263.

### Genomic localization of pre-miRNAs

**Figure 8:**
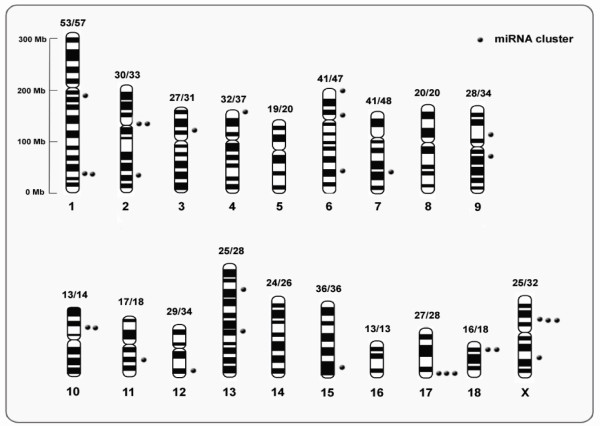
**Distribution of 30 miRNA clusters.** The number of base points near different bars indicates the number of clusters in the chromosome. The relative vertical dimension of the point on the bar represents the location of cluster. The label “number1 /number2” above every bar indicates the value of “pre-miRNAs/mature miRNA”.

To further establish the presence of miRNA precursors in the genome, all mature miRNAs (176 known and 315 novels) were mapped to the *S. scrofa* genome (Figure 8). As a result, 176 known mature miRNAs were mapped to 205 pre-miRNAs, and 315 novel miRNAs were mapped to 366 pre-miRNAs on the chromosomes. Our analysis revealed that the genomic density distribution of porcine milk pre-miRNAs (number of pre-miRNAs per Mb of each chromosome) was heterogeneous (Figure 8), ranging from 0.45 to 0.11 pre-miRNAs for 1 M of genomic sequence. Chromosomes with the highest and lowest densities of pre-miRNAs were chromosome 12 (29 pre-miRNAs per 64 Mbp) and chromosome 13 (25 pre-miRNAs per 219 Mbp), respectively. Interestingly, the medium-length X chromosome (144 Mbp, ranking 10th in length among the 19 chromosomes in pigs) was an exception by encoding an intermediate number (25 out of 366, 6.8%) of pre-miRNAs, corresponding to 0.17 pre-miRNAs for 1 M of genome sequence, but yet contained the most clusters.

In addition, we observed many mature miRNAs having multiple miRNA precursors located in the same or different chromosomes. Of the novel predicted miRNAs, 40 pre-miRNAs had two copies in the genome, 7 pre-miRNAs had 3 copies, 2 pre-miRNAs had 4 copies, 1 pre-miRNA had 8 copies and 249 pre-miRNAs were unique. With regard to known miRNAs, 4 pre-miRNAs had 3 copies, 22 pre-miRNAs had two copies and 149 pre-miRNAs had only one copy.

### Mature miRNAs

**Figure 9:**
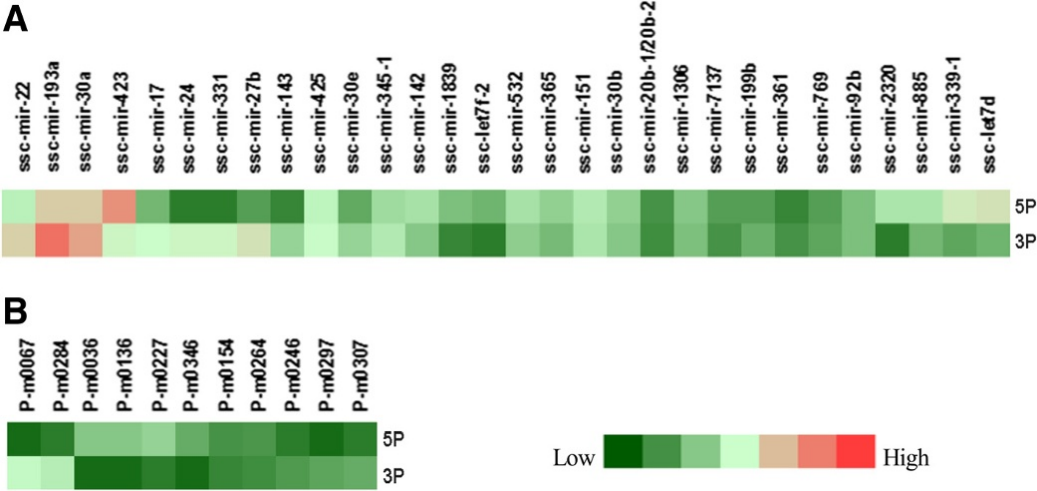
**5p and 3p arm expression of 46 pre-miRNAs. (A)** 30 known pre-miRNAs. **(B)** 11 novel pre-miRNAs (representing 15 coupled 3p and 5p arm sequences).

It is well accepted that only one of two strands generated from a precursor is preferentially incorporated into RNA-induced silencing complexes (RISC), whereas the complementary strand (miR*) may be degraded. Closer examination of mature miRNAs generated from pre-miRNAs showed that precursor miRNAs could be divided into three groups (Table 4): pre-miRNAs only with the left-arm sequence (5p), pre-miRNAs only with the right-arm sequence or both. Most pre-miRNAs seemed to be single-arm miRNAs (5p or 3p), while 50 pre-miRNAs possessed both 5p and 3p sequences (40 coupled mature miRNAs, all details were listed in Additional file 3: Table S3). Further analysis of the 40 coupled mature miRNAs indicated that most of the pre-miRNAs had no significant difference in abundance between the 5p-arm and 3p-arm sequences (Figure 9). Some miRNAs had different expression levels between the two strands. For example, ssc-miR-193a-3p had 67,154 counts, ranking first among all miRNAs, while ssc-miR-193a-5p had 2,538 counts. Conversely, miR-423-5p had 22,588 counts, while its complementary strand, miR-423-3p, had only 654 reads, and which was shared by miR-22, miR-30a, miR-339, miR-17, miR-24, miR-331, miR-27b and let-7d (detail in Additional file 3: Table S3).

**Table 4:** **Pre-miRNAs and their corresponding mature miRNAs type**

	Pre-miRNAs	miRNA-5p	miRNA-3p	Both	Mature miRNAs
known	205	67	57	33(26)#	176
novel	366	150	139	17(13)	315
total	571	217	196	50(39)	491

#Number in bracket represents mature miRNAs couple number.

**Figure 10:**
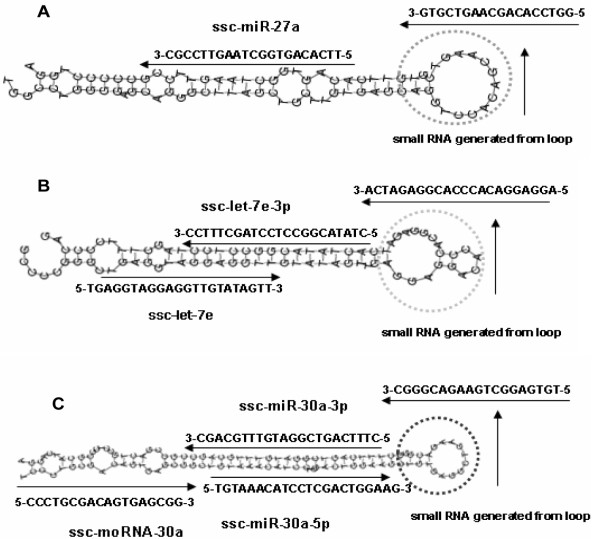
**Three distinctive pre-miRNAs identified in porcine milk exosomes. (A)** The ssc-miR-27a precursor produced a 3p-arm miRNA sequence and a loop-derived small RNA. **(B)** The ssc-let-7e precursor produced a 5p-arm sequence, a 3p-arm miRNA sequence and a loop-derived small RNA. **(C)** The ssc-miR-30a precursor produced a 5p-arm sequence, a 3p-arm miRNA sequence and a loop-derived small RNA. In addition, ssc-moRNA-3, belonging to new type of miRNA termed moRNA, was found at the 5’ end of pre-miR-30.

As described in other studies [51], some small RNAs were generated from the loop or the region between the loop and stem (ssc-let-7e, ssc-miR-27a and ssc-miR-30a) (Figure 10A–C). In addition, we detected another interesting type of small RNAs known as miRNA-offset RNAs, or “moRNAs”, which are derived from the ends of pre-miRNAs, predominantly from the 5’ end, independent of the mature miRNA. A good example of moRNAs and small RNAs generated from the loop was pre-miR-30a (Figure 10C). At the 5’ end of pre-miR-30, a 18 nt RNA sequence was found to be generated from the loop, downstream of ssc-miR-30a-5p (Figure 10C). The findings suggest that these RNAs may be only byproducts of Drosha and Dicer processing, or these small RNAs alternatively may take part in other important regulatory functions different from those of miRNAs.

### MiRNA clusters

**Figure 11:**
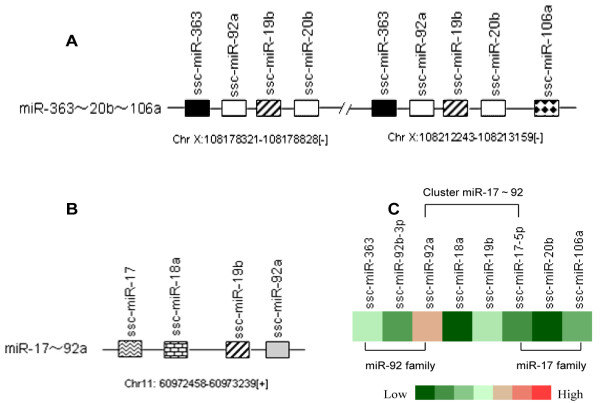
**MiR-363~20b~106a homologous or paralogous cluster and expression level. (A)** The miR-363/92a/19b/20b cluster and its homologous cluster miR-363/92a/19b/20b/106a on chromosome X were separated by a 33.5 kb DNA fragment. **(B)** The miR-17/18a/19/92a cluster was located on chromosome 11. In the genome, miR-92a/19b showed three copies; miR-363 and miR-20b had two copies; while miR-17, miR-18a and miR-106a had one copy. **(C)** Expression of mature miRNAs produced from the miR-363~20b~106a cluster and miR-17~92 cluster.

According to criteria for classification in miRbase, pre-miRNAs located on a chromosome with an interval of less than 10 kb are defined as belonging to an miRNA cluster. In our analysis, 30 (including 11 novel and 19 known) (Figure 8) clusters were detected (Table 5). Among all miRNAs clusters, there were several pre-miRNAs with intervening sequences of less than 1 kb, including 10 known clusters (miR-99b/let-7e/125a, miR-24-2/27b/23b, miR-99a/let-7c, miR-29b/29a, miR-221/222, miR-98/let-7f, miR-181c/d, miR-363/92a/19b-2/106a, miR-363/92a/19b-2, miR-181b-1/181a-1 and miR-17/18a/19b-1/92a-1) and 4 novel miRNAs clusters (cluster 3, 9, 12, 22). We identified a typical polycistronic miRNA cluster, miR-363/92a/19b-2/20b, on chromosome X. Interestingly, the homologous cluster, miR-363/92a/19b-2/20b/106a on chromosome X, was located 33.5 kb downstream of miR-363/92a/19b-2/20b (Figure 11A, C), and a paraologous cluster miR-17/18a/19b-1/92a-1 was found on chromosome 11 (Figure 11B, C). The organization of miRNA precursors in the genome may account for variable levels of expression and regulation of mature miRNAs.

**Table 5:** **miRNAs cluster**

Cluster No.	Cluster name	members	chromosome
cluster 1	new	p-m0165, p-m0166	1
cluster 2	mir-181a-2/181b-2	ssc-mir-181a-2, ssc-mir-181b-2	1
cluster 3	new	ssc-miR-199b, P-m0179	1
cluster 4	miR-24-1/27a/23a	ssc-mir-24-1, ssc-miR-27a, ssc-mir-23a	2
cluster 5	miR-181c/d	ssc-miR-181c, ssc-mir-181d	2
cluster 6	miR-143/145	ssc-mir-143, ssc-miR-145	2
cluster 7	let-7a/let-7f-2/let-7d	ssc-let-7a-2, P-m0204, ssc-let-7f-2, ssc-let-7d	3
cluster 8	miR-30d/30b	ssc-mir-30d, ssc-mir-30b	4
cluster 9	new	P-m0263, P-m0264	6
cluster 10	mir-99b/let-7e/125a	ssc-mir-99b, ssc-let-7e, ssc-mir-125a	6
cluster 11	mir-30c-1/ mir-30e	ssc-mir-30c-1,ssc-mir-30e	6
Cluster 12	new	P-m0302,P-m0318	7
cluster 13	let-7a-1/miR-100	ssc-let-7a-1, ssc-mir-100	9
cluster 14	new	P-m0346, P-m0347, P-m0348,P-m0349	9
cluster 15	miR-181b-1/181a-1	ssc-mir-181b-1, ssc-mir-181a-1	10
cluster 16	miR-24-2/27b/23b	ssc-mir-24-2, ssc-mir-27b, ssc-mir-23b	10
cluster 17	miR-17-92a-1	ssc-mir-17, ssc-mir-18a,ssc-mir-19b-1, ssc-mir-92a-1	11
cluster 18	new	P-m0024, P-m0025, P-m0026	12
cluster 19	miR-425/191	ssc-mir-425, ssc-mir-191	13
cluster 20	miR-99a/let-7c	ssc-mir-99a, ssc-let-7c	13
cluster 21	new	P-m0099, P-m0083	15
cluster 22	new	P-m0124, P-m0113	17
cluster 23	new	P-m0125, P-m0126, P-m0127, P-m0128, P-m0114	17
cluster 24	new	P-m0130, P-m0131	17
cluster 25	miR-29b-1/29a	ssc-mir-29b-1, ssc-mir-29a	18
cluster 26	new	P-m0136, P-m0137, ssc-mir-183	18
cluster 27	miR-221/222	ssc-mir-221, ssc-mir-222	X
cluster 28	miR-98/let-7f-1	ssc-mir-98, ssc-let-7f-1	X
cluster 29	miR-363~20b	ssc-mir-363-1, ssc-mir-92a-2, ssc-mir-19b-2, P-m0370, sc-mir-20b-1/ssc-mir-20b-2	X
Cluster 30	miR-363~20b	ssc-mir-363-1, ssc-mir-92a-2, ssc-mir-19b-2, P-m0371, ssc-mir-20b-1/ssc-mir-20b-2, ssc-miR-106a	X

### MiRNA families

**Figure 12:**
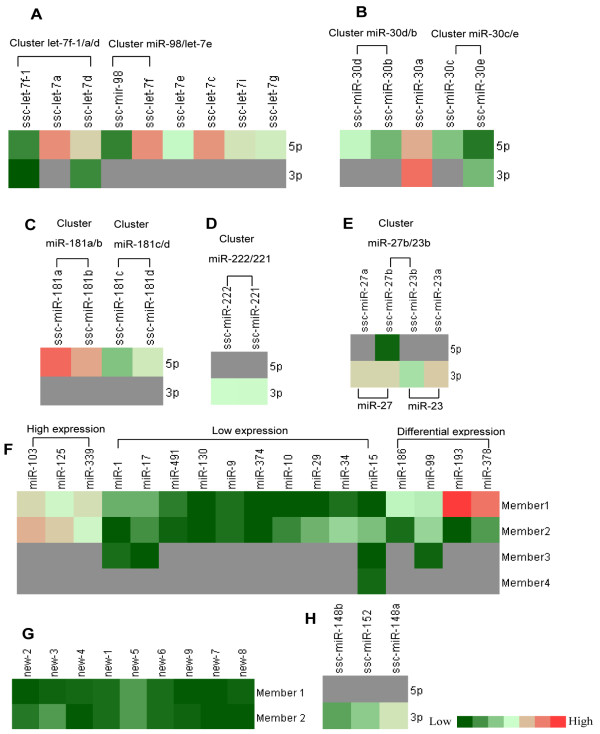
**Expression of miRNA families. (A)** Expression of the let-7f family; let-7f-1, let-7a and let-7d formed a cluster, while miR-98 and let-7e formed a cluster. **(B)** Expression of miR-30 family; miR-30d clustered with miR-30b, and miR-30c clustered with miR-30e. **(C)** Expression of miR-181 family; miR-181a clustered with miR-181b, and miR-181c clustered with miR-181d. **(D)** MiR-222 and miR-221; miR-222 and miR-221 belonged to the same family and the same cluster. **(E)** MiR-27 family and miR-23 family; miR-27b and miR-23b formed a cluster in the genome. **(F)** Expression and clustering of remaining miRNA families; members 1, 2, 3 and 4 represent members of the indicated family. **(G)** Novel miRNA family with low expression. **(H)** Expression of the miR-148 family containing milk “marker” miRNAs.

It is widely believed that the members of a given miRNA family regulate very similar sets of target genes. Apart from miRNA clusters, miRNA families were also recognized in the miRNAs of exosomes. Based on seed sequences, 35 miRNA families were identified (26 known and 9 novel miRNA families) to contain at least two members and the identification of novel miRNAs added new members to 5 known families (Table 6). In our study, 8 miRNA families (let-7, mir-1, mir-17, mir-181, mir-148, mir-30, mir-92 and mir-99) were found with at least 3 members among all exosome miRNAs. The let-7 family had 9 members, miR-181 family had 4 members (miR-181a/b/c/d) and miR-30 family had 5 members (miR-30a/b/c/d/e). Most importantly, these miRNAs were highly expressed, and let-7 family members (let-7a/f/c), miR-181a and miR-30a-3p were enriched among the top 10 miRNAs. However, members in the same family were highly differentially expressed. In the miR-181 family, miR-181a and miR-181b were dominant types with 13,345 reads and 3,333 reads, respectively. Similarly, miR-30a was the most abundant in the miR-30 family. The differential expression of members in the same family may be partly due to regulation of their precursors [52]. On the other hand, combined with the cluster analysis, we also observed that some miRNAs shared not only the same cluster but also the same families. These miRNAs included 181a/b, let-7f/miR-98, 181c/d, let-7a/f-5p/d-5p, 30b/d, 30c/e and miR-221/222. More interestingly, family members of the same cluster seemed to share expression patterns (Figure 12A–E). As mentioned above, miR-17-5p, miR-363, miR-106a, miR-18a, miR-19b, miR-92a, miR-20b and miR-92b formed a complex cluster and family network, and they also showed different expression patterns. MiR-92a, miR-19b and miR-363 were found to be highly expressed, while miR-17-5p, miR-18a, miR-20b and miR-106a were lowly expressed. The difference in abundance of the homologous or paralogous clusters may be attributed to the copy number of miRNA precursor itself or to the post-transcriptional regulation of the process of generating a mature miRNA from the precursor miRNA.

**Table 6:** **miRNAs family**

	Family	Seed	Number	Members
Family 1	let-7	GAGGTAG	9	ssc-let-7i, ssc-miR-98, ssc-let-7a,ssc-let-7f, ssc-let-7c, ssc-let-7 g, ssc-let-7e, ssc-let7d-5p
Family 2	mir-1	GGAATGT	3	ssc-miR-1, ssc-miR-206, PC-117
Family 3	mir-10	ACCCTGT	2	ssc-miR-10b, ssc-miR-10a
Family 4	mir-103	GCAGCAT	2	ssc-miR-107, ssc-miR-103
Family 5	mir-125	CCCTGAG	2	ssc-miR-125a, ssc-miR-125b
Family 6	mir-130	AGTGCAA	2	ssc-miR-130b, ssc-miR-130a
Family 7	mir-148	CAGTGCA	3	ssc-miR-148b, ssc-miR-152, ssc-miR-148a
Family 8	mir-17	AAAGTGC	3	ssc-miR-106a, ssc-miR-17-5p, ssc-mir-20b-1/ssc-mir-20b-2
Family 9	mir-181	ACATTCA	4	ssc-miR-181a, ssc-miR-181b, ssc-miR-181c, ssc-miR-181d-5p
Family 10	mir-186	AAAGAAT	2	ssc-miR-186, PC-36
Family 11	mir-193	ACTGGCC	2	ssc-miR-193a-3p, PC-3
Family 12	mir-221	GCTACAT	2	ssc-miR-222, ssc-miR-221
Family 13	mir-23	TCACATT	2	ssc-miR-23b, ssc-miR-23a
Family 14	mir-27	TCACAGT	2	ssc-miR-27a,ssc-miR-27b
Family 15	mir-29	AGCACCA	2	ssc-miR-29b, ssc-miR-29c
Family 16	mir-30	GTAAACA	5	ssc-miR-30e-5p, ssc-miR-30c, ssc-miR-30d, ssc-miR-30b-5p, ssc-miR-30a-5p
Family 17	mir-30(#)	TTTCAGT	2	ssc-miR-30a-3p, ssc-miR-30e-3p
Family 18	mir-339	CCCTGTC	2	ssc-miR-339-5p, ssc-miR-4334-3p
Family 19	mir-34	GGCAGTG	2	ssc-miR-34c, ssc-miR-34a,
Family 20	mir-363	ATTGCAC	3	ssc-miR-363, ssc-miR-92b-3p, ssc-miR-92a
Family 21	mir-374	TATAATA	2	ssc-miR-374b-5p, ssc-miR-374a,
Family 22	mir-378	CTGGACT	2	ssc-miR-378, PS-2
Family 23	mir-491	GTGGGGA	2	ssc-miR-491, PC-122
Family 24	mir-497	AGCAGCA	4	ssc-miR-497, ssc-miR-15b, ssc-miR-16, ssc-miR-15a
Family 25	mir-9	CTTTGGT	2	ssc-miR-9-2, ssc-miR-9-1,
Family 26	mir-99	ACCCGTA	3	ssc-miR-99a, ssc-miR-99b, ssc-miR-100
Family 27	new-1	CATGATT	2	P-m0040-3p(PC-9), P-m0240-5p(PC-232),
Family 28	new-2	AGAGGGA	2	P-m0048-3p(PC-152), P-m0064-3p(PC-217)
Family 29	new-3	ATTTGAT	2	P-m0084-5p(PC-228), P-m0105-3p(PC-72),
Family 30	new-4	GCTAGGA	2	P-m0110-5p(PC-112), P-m0139-5p(PC-111)
Family 31	new-5	TATGGAT	2	P-m0113-3p(PC-130), P-m0129-5p(PC-129)
Family 32	new-6	CCTGGAT	2	P-m0142-5p(PC-164), P-m0179-3p(PC-165)
Family 33	new-7	CATATTT	2	P-m0161-3p(PC-231), P-m0244-3p(PC-162)
Family 34	new-8	GTTTGGA	2	P-m0183-3p(PC-286), P-m0325-5p(PC-128)
Family 35	new-9	CTTTGGG	2	P-m0229-5p(PC-15), P-m0342-3p(PC-105)

The underline indicated this family contains novel miRNAs. #: due to miRNAs classification by seed sequence, 3p and 5p of miR-30 represent different miRNAs families. PC is unique ID for porcine miRNAs candidate.

In addition, many miRNA families showed low expression (count number <100) in milk exosomes, such as the miR-1, miR-130, miR-17, miR-10, miR-29, miR-374, mir-9, miR-15 and miR-491 families (Figure 12F), which are routinely expressed in specific tissues [53–56]. Interestingly, all 9 novel miRNAs families showed extremely low expression levels (Figure 12G, count number <50), indicating that these miRNAs may only be expressed in certain physiology processes and may be the reason for why these miRNAs have not been detected until now. MiR-148a was reported to be an important biomarker for milk exosome miRNAs [28, 57]. In this study, three members of this family (miR-148a, miR-148b and miR-152, Figure 12H) showed modest expression levels, suggesting that miR-148 may be a stably expressed miRNA in exosomes of most mammals including pigs.

### GO and KEGG pathway analyses

To better understand the role of miRNAs in pig milk exosomes, potential targets of miRNAs at the pig genome level were explored by using our previous method [58]. We selected the top 10 miRNAs for target prediction using RNAhybrid, which found 2,333 potential transcripts. GO analysis showed that those targets were enriched in many processes (Table 7), including antigen processing and presentation, MHC class II protein complex and transcription. Pathways analysis revealed these targets were enriched in 7 KEGG pathways (Table 8), which are essential for piglet metabolism (glycerophospholipid metabolism and citrate cycle), immunity (cell adhesion molecules, asthma and intestinal immune network for IgA production, antigen processing and presentation) and development (Notch signaling pathway). The results suggest that these milk miRNAs likely take part in regulation of important protective functions in piglets, including the intestinal immune network for IgA production and antigen processing and presentation.

**Table 7:** **Gene ontology analysis of potential targets of top10 miRNAs**

Category	Term	Count	P-Value
Biological process	transcription	15	6.70E-03
Biological process	regulation of transcription	21	1.00E-02
Biological process	antigen processing and presentation	7	1.50E-02
Biological process	regulation of RNA metabolic process	17	1.70E-02
Biological process	regulation of transcription, DNA-dependent	17	1.70E-02
Biological process	antigen processing and presentation of peptide or polysaccharide antigen via MHC class II	4	2.20E-02
Cellular Component	MHC class II protein complex	6	3.70E-04
Cellular Component	MHC protein complex	7	5.00E-03
Cellular Component	large ribosomal subunit	3	2.90E-02
Molecular Function	transcription regulator activity	20	1.80E-03
Molecular Function	sequence-specific DNA binding	13	2.60E-03
Molecular Function	ligand-dependent nuclear receptor activity	7	5.80E-03
Molecular Function	transcription factor activity	15	1.20E-02
Molecular Function	steroid hormone receptor activity	6	2.10E-02
Molecular Function	DNA binding	20	2.40E-02
Molecular Function	phosphatase regulator activity	4	2.90E-02
Molecular Function	protein phosphatase regulator activity	4	2.90E-02

**Table 8:** **KEGG pathway analysis of potential targets of top10 miRNAs**

Term	Count	gene	p-value
ssc04514: Cell adhesion molecules (CAMs)	14	CADM3, CD4, CD40, F11R, LOC100521555, SELE, SELL, SELP, SLA, SLA-DMA, SLA-DOA, SLA-DOB, SLA-DRA, SLA-DRB1	1.65E-03
ssc05310: Asthma	7	CD40, SLA, SLA-DMA, SLA-DOA, SLA-DOB, SLA-DRA, SLA-DRB1	5.55E-03
ssc04672: Intestinal immune network for IgA production	8	CD40, SLA, SLA-DMA, SLA-DOA, SLA-DOB, SLA-DRA, SLA-DRB1, TGFB3	2.15 E-02
ssc04330: Notch signaling pathway	5	DLL4, PTCRA, LOC733643, APH1A, NOTCH4	2.33E-02
ssc04612: Antigen processing and presentation	10	CD4, LOC100152370, NFYB, PSME1, SLA, SLA-DMA, SLA-DOA, SLA-DOB, SLA-DRA, SLA-DRB1	3.00E-02
ssc00564:Glycerophospholipid metabolism	6	AGPAT1, AGPAT4, |AGPAT6, GNPAT, LOC100152491, PCYT1B	4.25E-02
ssc00020:Citrate cycle (TCA cycle)	5	ACO2,DLST, IDH2, LOC100157889, PCK1	4.57E-02

### Targets of miRNAs in IgA immune network

**Figure 13:**
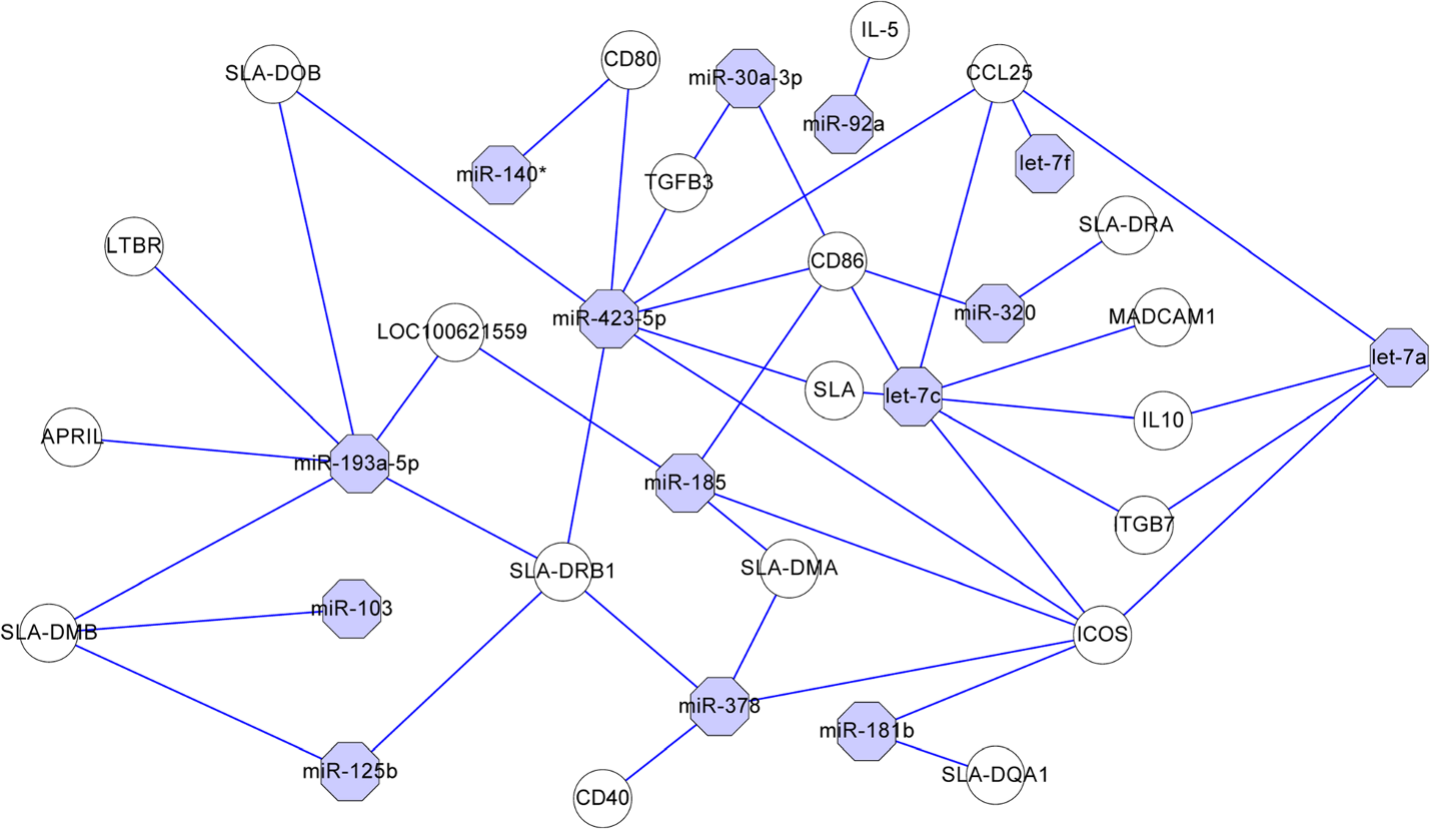
**MiRNAs targeting the IgA immune network.** The top 20 miRNAs were analyzed, and 14 of them were found to participate in the IgA immune network, involving 20 target genes. Different colors and shapes represent various relationships between miRNAs and genes.

As shown by GO and KEGG analysis, many of the identified miRNAs were predicted to participate in immunity, similar to findings of other studies [28]. To further understand those miRNAs, the top 20 predicted miRNAs were selected for in-depth analysis of the IgA immune network. The results showed that 14 of the top 20 miRNAs are likely involved in the IgA network and may target about 20 immune-related genes (*APRIL, CCL25, CD40, CD80, CD86, ICOS, IL10, IL-5, ITGB7, LOC100621559, LTBR, MADCAM1, SLA, SLA-DMA, SLA-DMB, SLA-DOB, SLA-DQA1, SLA-DRA, SLA-DRB1 and TGFB3*) (Figure 13). These results suggest that porcine milk miRNAs take part in regulation of the IgA network and immunity of piglets. In addition, several miRNAs shared the same target gene. Interestingly, all of the let-7 family members (let-7a, let-7c and let-7f) could target *CCL25*. Those miRNAs are proposed to play a key role in IgA production in the piglet digestive tract and deserve further exploration, as mucosal immunity is critically important for the protection of newborn piglets.

## Discussion

In the present study, a comprehensive miRNA expression profile of porcine breast milk exosomes was explored via a deep sequencing approach. We found in total 176 known miRNAs (miRBase 20.0) and 366 pre-miRNAs producing 315 mature miRNAs. Luciferase reporter assay was used to explore the targets of two predicted novel miRNAs in this study. Results indicated both of them down-regulated the luciferase expression by targeting 3’UTR of IGF-1R. All these pre-miRNAs were distributed in 30 clusters (11 novel and 19 known clusters), and the mature miRNAs could be assigned to 35 families (26 known and 9 unknown families). GO and KEGG pathway analyses show that those miRNAs may participate in many different immune-related processes. An analysis of the top 20 miRNAs showed that 14 of them may be involved in many regulatory aspects of the IgA immune network.

A recent study of exosome miRNAs in Yorkshire sow milk discovered 180 pre-miRNAs, including 140 known porcine pre-miRNAs and 40 novel pre-miRNAs, which encode 237 mature miRNAs (234 unique miRNAs) [29] In the current study, we discovered 205 known porcine pre-miRNAs (176 mature miRNAs) and 366 novel pre-miRNAs (315 mature miRNAs), approximately 254 more mature miRNAs than were revealed in the former report [29]. Therefore, our results substantially supplement the known pig miRNAs, particularly milk exosome miRNAs. Interestingly, most of the novel miRNAs were low in abundance (312 miRNAs with less than 100 reads and only 3 miRNAs with >100 reads), which is possibly the reason for why these miRNAs were not detected in a previous study by Gu *et al.*[29]. Further comparison revealed that miR-191 and let-7a, which potentially play a vital role in immunity, were found in both that study and in the top 10 miRNAs of our study. Other miRNAs identified previously (miR-30a-5p, miR-25-3p, miR-182-5p, miR-200c-3p and miR-375-3p) were not detected in our study. Furthermore, miR-148a, a potential biomarker for quality control in bovine milk [57] and human milk [28], which was found to be highly expressed throughout the lactation period of Yorkshire sows [29], was only detected at a moderate level in Landrace pigs in our study. MiR-148a has been reported to be a tumor metastasis suppressor in gastric cancer [59], and ectopic expression of miR-148a was shown to induce apoptosis and silence *Bcl-2* in colorectal cancer cells [60]. By bioinformatics analysis, miR-148a was determined to be possibly related to immunity and gastrointestinal health, but the underlying regulatory mechanism remains unclear.

MiR-92a belongs to the miR-17 ~92 cluster with seven miRNAs (miR-17-5p, miR-17-3p, miR-18a, miR-19a, miR-19b, miR-20a and miR-92a) and was first described as an oncogenic miRNA cluster involved in B-cell lymphoma [61]. Recent studies indicated that the miRNA-17-92 (miR-17-92) cluster directly targets the *TGFB* pathway in cancer cell lines in the mouse embryo stage [62]. In addition, the miR-17-92 cluster also participates in normal development of the heart, lungs and immune system [63]. MiR-19 can promote leukemogenesis in Notch1-induced T-cell acute lymphoblastic leukemia (T-ALL) *in vivo*[64]. Overexpression of the mir-17–mir-18a–mir-19b-1 cluster was shown to accelerate Myc-induced tumor development in a mouse B-cell lymphoma model [61]. The combined results above imply that members of the cluster miR-363/92a/19b-2/20b/106a may be related to cell proliferation and development. In porcine milk, miR-363/92a/19b-2/20b (miR-363/92a/19b-2/20b/106a) and miR-17/18a/19b-1/92-1 were also detected. The miR-181 (181a/b/c/d) family is related to the development of different cells. It was reported that miR-181c/d can inhibit cell cycle and proliferation and that miR-181c regulates *TNF-α*[65]. The miR-30(b/c/d/e) family regulates kidney development by targeting the transcription factor *Xlim1/Lhx1* in *Xenopus*[66]. The well-known let-7(a/b/d/f) family is involved in oncogene expression [67], and let-7/miR-98 family members are expressed late in mammalian embryonic development [68]. Thus, these miRNAs mentioned above may participate in development of the piglet digestive tract.

Notably, some miRNAs among the top 10 identified here have been reported to be related to immunity (miR-320, miR-181a, miR-30a-3p, let-7a, let-7f and let-7c) and development (miR-193a-3p, miR-378 and miR-191). MiR-193a-3p was demonstrated to regulate cell proliferation, cell cycle progression *in vitro* and in nude mice [69]. MiR-378 promotes osteoblast differentiation by targeting polypeptide N-acetylgalactosaminyltransferase 7 (*GalNAc-T7* or *GalNT7*) [70], and miR-191 regulates erythroid differentiation in mammals by up-regulating erythroid-enriched genes *Riok3* and *Mxi1*[71]. Meanwhile, miR-320 is able to inhibit HL-60 cell proliferation by suppressing receptor 1 (*TfR-1; CD71*) [72], and miR-181a was believed to act as an intrinsic antigen sensitivity “rheostat” during T cell development [73]. MiR-320, miR-181a, miR-30a-3p and let-7 were shown to be downregulated in colorectal cancer [74]. Of course, further experimental evidence is needed to verify that these miRNAs are indeed related to immunity of the piglet digestive tract.

IgA is a major immunoglobulin in milk [75]. Expression of the polymeric IgA receptor (*pIgR*) in mammary epithelial cells contribute much to the development of the immune system at the early stage of lactation [76]. In the present study, some miRNAs were predicted to target genes (*CD40, SLA, SLA-DMA, SLA-DOA, SLA-DOB, SLA-DRA, SLA-DRB1 and TGFB3*) involved in processes of the intestinal immune network for IgA production in porcine milk. *CD40* is a B-cell antigen activated during immune responses [77]. *CD40* and CD40 ligand (*CD40L*) expressed on activated T cells are essential to B cell proliferation [78] and secretion of IgG, IgA and IgE [79]. SLA Class I were found to be expressed in the epithelial and lamina propria cells of the intestine in adult pigs and to be involved in mother-newborn interactions [80]. A study in humans showed that *TGF-β* acts as a specific switch for IgA present at early stages of development of B cells [81].

In the present study, the top 20 miRNAs were used for IgA network analysis. *APRIL* was the predicted target of miR-193a-5p, which is essential to triggering IgA_2_ class switch in human B cells. Intestinal epithelial cells (IECs) release *APRIL* after sensing bacteria through Toll-like receptors, and mucosal vaccines activate IECs to induce more effective IgA_2_ responses [82]. The let7 family and miR-423-5p were predicted to target *CCL25*, a potent and selective chemoattractant for IgA antibody-secreting cells [83]. *CCL25* is known to selectively modulate immune responses, specifically the localization of T lymphocytes to the small-intestinal mucosa [84]. *CD80* and *CD86*, which are costimulators of T lymphocytes [85], were identified as possible targets of five miRNAs in our study. Let-7a, let-7c, miR-181b, miR-185, miR-378 and miR-423-5p were predicted to target the inducible co-stimulatory molecule (*ICOS*), which plays a key role in regulating T-cell differentiation, T-cell proliferation, and secretion of lymphokines, providing effective help for antibody secretion by B cells [86]. We hypothesize that some miRNAs identified here in porcine milk regulate IgA production in the intestine of piglets, which may play an important role in mucosa immunity. However, their regulatory mechanisms warrant further study.

## Conclusions

In conclusion, the present study revealed 176 known miRNAs and 366 (315 mature miRNAs) novel pre-miRNAs in porcine milk, most of which were predicted to be involved in regulation of digestive tract development and immunity of newborn piglets. These findings contribute to an increased understanding of the roles of miRNAs in porcine (*S. scrofa)* milk exosomes and to building the foundation for understanding their physiological functions and regulatory mechanisms.

## Availability of supporting data

All the supporting data has been deposited in https://mynotebook.labarchives.com/share/allinchen/MTkuNXwxMzMxMS8xNS0yL1RyZWVOb2RlLzE1NzEyODU2fDQ5LjU= with a DOI:10.6070/H4DN432G.

## Electronic supplementary material

Additional file 1: Table S1: 176 known mature miRNAs.xls. (XLS 109 KB)

Additional file 2: Table S2: 315 novel miRNAs.xls. (XLS 196 KB)

Additional file 3: Table S3: Pre-miRNAs with 3p and 5p sequence.xls. (XLS 77 KB)
